# Conformation of HIV-1 Envelope Governs Rhesus CD4 Usage and Simian-Human Immunodeficiency Virus Replication

**DOI:** 10.1128/mbio.02752-21

**Published:** 2022-01-11

**Authors:** Geraldine Vilmen, Anna C. Smith, Hector Cervera Benet, Rajni Kant Shukla, Ross C. Larue, Alon Herschhorn, Amit Sharma

**Affiliations:** a Department of Veterinary Biosciences, The Ohio State University, Columbus, Ohio, USA; b Department of Microbial Infection & Immunity, The Ohio State University, Columbus, Ohio, USA; c Division of Infectious Diseases and International Medicine, Department of Medicine, University of Minnesota, Minneapolis, Minnesota, USA; d Department of Cancer Biology and Genetics, The Ohio State University, Columbus, Ohio, USA; Yale University; Rutgers-Robert Wood Johnson Medical School

**Keywords:** HIV-1 envelope, CD4, rhesus macaque, SHIV, conformation, viral entry

## Abstract

Infection of rhesus macaques with simian-human immunodeficiency viruses (SHIVs) is the preferred model system for vaccine development because SHIVs encode human immunodeficiency virus type 1 (HIV-1) envelope glycoproteins (Envs)—a key target of HIV-1 neutralizing antibodies. Since the goal of vaccines is to prevent new infections, SHIVs encoding circulating HIV-1 Env are desired as challenge viruses. Development of such biologically relevant SHIVs has been challenging, as they fail to infect rhesus macaques, mainly because most circulating HIV-1 Envs do not use rhesus CD4 (rhCD4) receptor for viral entry. Most primary HIV-1 Envs exist in a closed conformation and occasionally transit to a downstream, open conformation through an obligate intermediate conformation. Here, we provide genetic evidence that open Env conformations can overcome the rhCD4 entry barrier and increase replication of SHIVs in rhesus lymphocytes. Consistent with prior studies, we found that circulating HIV-1 Envs do not use rhCD4 efficiently for viral entry. However, by using HIV-1 Envs with single amino acid substitutions that alter their conformational state, we found that transitions to intermediate and open Env conformations allow usage of physiological levels of rhCD4 for viral entry. We engineered these single amino acid substitutions in the transmitted/founder HIV-1_BG505_ Envs encoded by SHIV-BG505 and found that open Env conformation enhances SHIV replication in rhesus lymphocytes. Lastly, CD4-mediated SHIV pulldown, sensitivity to soluble CD4, and fusogenicity assays indicated that open Env conformation promotes efficient rhCD4 binding and viral-host membrane fusion. These findings identify the conformational state of HIV-1 Env as a major determinant for rhCD4 usage, viral fusion, and SHIV replication.

## INTRODUCTION

The ability of viruses to efficiently utilize orthologous entry receptors determines which species can be infected, and this principle is often exploited for developing animal models of viral infection. Rhesus macaques serve as critical animal model for preclinical human immunodeficiency virus type 1 (HIV-1) research. However, macaque models are limited by the fact that HIV-1 does not persistently infect macaques. Chimeric simian-human immunodeficiency viruses (SHIVs), constructed by replacing simian immunodeficiency virus (SIV) envelope glycoproteins (Envs) with that from HIV-1, have been developed as surrogates to study HIV-1 infection in rhesus macaques (1). Development of SHIVs has been a challenging process, mainly because most Envs from circulating HIV-1 variants do not use rhesus CD4 (rhCD4) efficiently for viral entry, and therefore, SHIVs replicate poorly in rhesus lymphocytes and do not establish persistent infection (2). Existing pathogenic SHIVs are a highly selected subset of viruses because they encode Envs from lab-adapted and chronic-stage HIV-1 variants, which can mediate entry using rhCD4. Moreover, to increase their pathogenicity, SHIVs generally require extensive adaptation to macaques (3–8), at least in part to optimize rhCD4-mediated entry (9). However, adaptation alters the antigenic features of Envs (10), limiting the translational utility of adapted SHIVs.

Binding of the metastable HIV-1 Env trimer to CD4 triggers a series of conformational changes in Env; i.e., the Env transitions from a functionally “closed” (state 1) to an “intermediate” (state 2) to an “open” (state 3) conformation (11–13). Unliganded Env trimers of most primary isolates only infrequently transit from closed to downstream, more open states (11, 14). The conformational dynamics of HIV-1 Envs can affect viral tropism by allowing adaptability to cells with different CD4 levels. For example, HIV-1 Envs that frequently sample state 2 and state 3 conformations can more efficiently infect cells that express low levels of human CD4 (huCD4) (15, 16). Importantly, the ability of HIV-1 Envs to utilize low levels of huCD4 positively correlates with their ability to utilize rhCD4 (17). Based on these insights, we hypothesized that open conformational states of HIV-1 Envs can overcome the rhCD4 entry barrier, which can, in turn, increase replication of SHIVs in rhesus lymphocytes. In this study, we generated HIV-1 Envs and SHIVs with single amino acid substitutions that alter Env conformational state. Our results show that SHIVs with open Env conformation bind and utilize rhCD4 efficiently and display higher viral fusion and replication in rhesus lymphocytes.

## RESULTS

### T/F and early-stage HIV-1 Envs do not use physiological levels of rhCD4 efficiently for viral entry.

**FIG 1:**
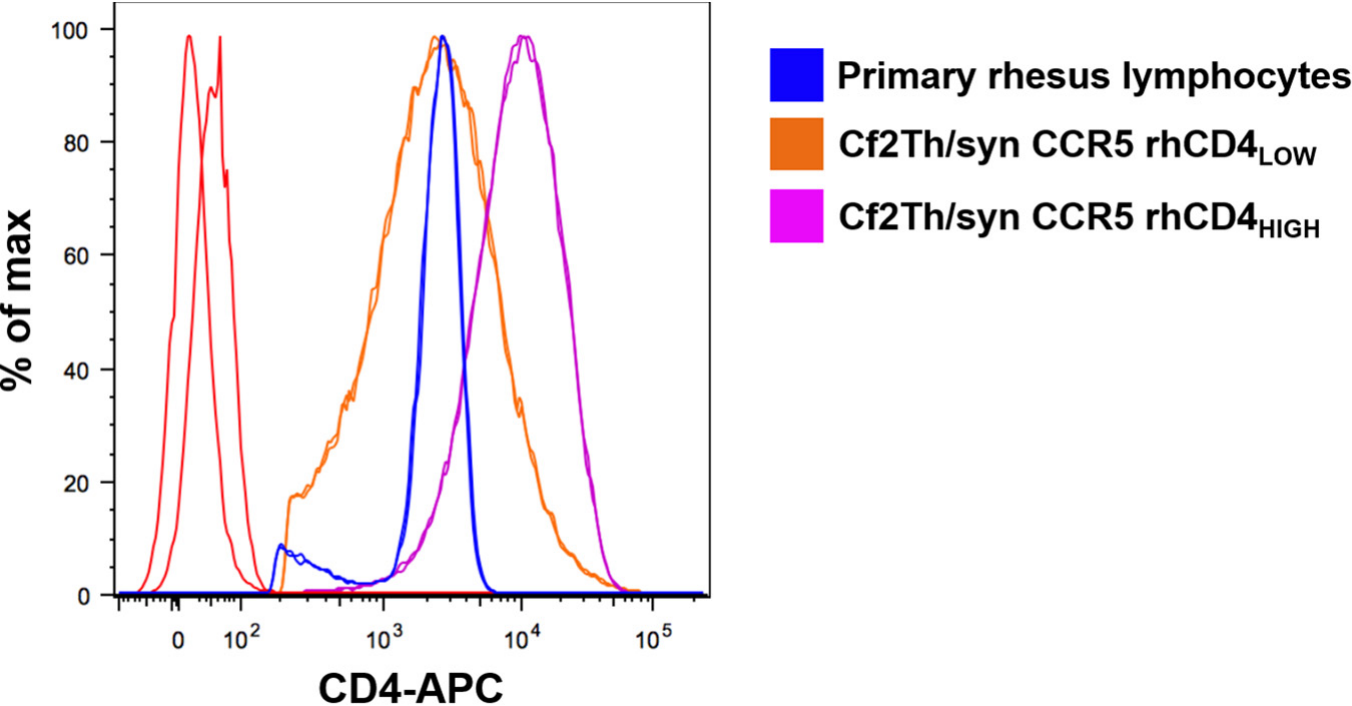
Expression levels of rhCD4 stably introduced into Cf2Th/syn CCR5 cells. Shown are expression levels of CD4 on primary rhesus macaque lymphocytes and rhCD4_LOW_ and rhCD4_HIGH_ Cf2Th/syn CCR5 cell lines as measured by flow cytometry using an APC-conjugated anti-CD4 antibody. The histograms represent the expression of CD4 on the cell surface. The data are representative of those from two independent experiments.

**FIG 2:**
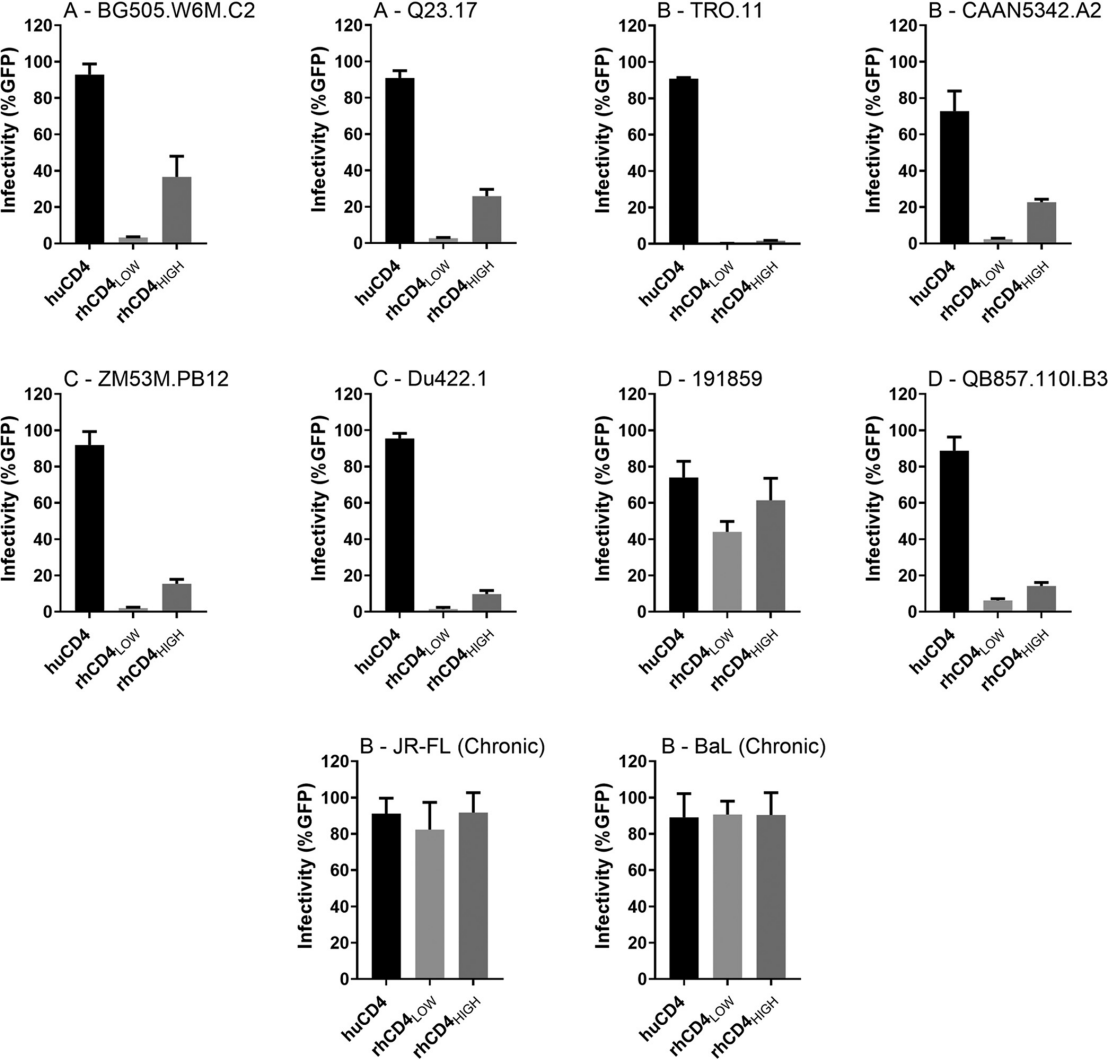
Ability of transmitted/founder and early-stage HIV-1 Envs to use physiological levels of rhCD4 for viral entry. Cf2Th/syn CCR5 cells expressing human or rhesus CD4 (indicated along the *x* axis) were infected with HIV-1_Q23ΔEnvGFP_ pseudotyped with indicated Env clones. Infection was measured by flow cytometry as percentage of GFP-positive cells 48 h postinfection. Graphs show percentage of infected cells for indicated Env at a multiplicity of infection of 1. Env clones are labeled at the top, with first letter indicating the subtype. Bars represent the averages from three independent experiments. Error bars represent standard deviations.

Envs of most circulating HIV-1 isolates from early stages of infection, including the transmitted/founder (T/F) variants, do not use rhCD4 efficiently for viral entry (17). To test our hypothesis that open Env conformations can overcome the rhCD4 entry barrier and mediate entry using physiological levels of rhCD4 expressed on rhesus lymphocytes, we first generated Cf2Th/syn CCR5 cells that stably express either low or high levels of rhCD4 (rhCD4_LOW_ and rhCD4_HIGH_). Importantly, we sorted and selected the rhCD4_LOW_ cells such that their CD4 expression levels were representative of primary rhesus lymphocytes (Fig. 1). The cells were then infected with green fluorescent protein (GFP)-reporter HIV-1 pseudotyped with Envs that were obtained from HIV-1 isolates from acute/early stages of infection, including the T/F isolates (Table 1). As a control, Cf2Th/syn CCR5 cells expressing huCD4 were also infected and facilitated entry by all of these Envs (Fig. 2). In contrast to huCD4-mediated entry, rhCD4_LOW_ cells did not facilitate efficient entry by seven out of eight Envs. Even with rhCD4_HIGH_ cells, which express high levels of rhCD4, viral entry was much lower (∼2.5- to 53-fold) than for huCD4. Only subtype D Env 191859 was able to gain entry into rhCD4_LOW_ cells, but it was still ∼1.7-fold lower than huCD4, highlighting that there are some rare primary Env that can inherently utilize rhCD4. As positive controls, two Envs from chronic-stage HIV-1 isolates (JR-FL and BaL) were tested and were able to gain entry using both huCD4 and rhCD4—consistent with the ability of Envs from lab-adapted and chronic-stage isolates to utilize rhCD4 for entry (17).

**TABLE 1:** Transmitted/founder and early-stage Env clones used in this study

Envelope clone	Subtype	Mode of natural transmission^*a*^	Source^*b*^	GenBank accession no.	Time postinfection (wks)
BG505.W6M.C2	A	M-C	PBMC	DQ208458	6
Q23.17	A	M-F	PBMC	AF004885	11
TRO.11	B	M-M	ccPBMC	AY835445	4
CAAN5342.A2	B	M-M	Plasma	AY835452	<12
Du422.1	C	M-F	ccPBMC	DQ411854	8
ZM53M.PB12	C	F-M	PBMC	AY423984	<12
QB857.110I.B3	D	M-F	PBMC	FJ866138	16
191859	D	M-F	Plasma	JX203064	Fiebig I

a M-C, mother to child; M-F, male to female; M-M, male to male; F-M, female to male.

b PBMC, Env cloned from uncultured PBMCs isolated directly from patient; ccPBMC, patient PBMCs (or virus from these PBMCs) underwent short-term coculture with PBMCs from HIV-1-negative donors to amplify virus before cloning; Plasma, Env cloned from virion RNA in plasma isolated directly from patient.

### Transitions to intermediate and open Env conformations allow usage of rhCD4 for viral entry.

**FIG 3:**
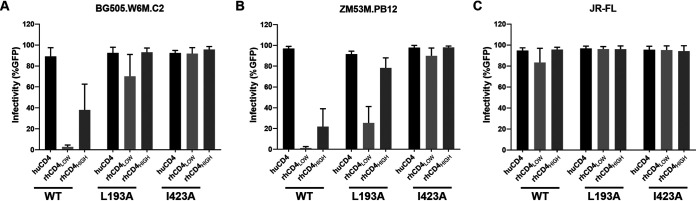
Effect of Env conformation on usage of rhCD4 for viral entry. Cf2Th/syn CCR5 cells expressing human or rhesus CD4 (indicated along the *x* axis) were infected with HIV-1_Q23ΔEnvGFP_ pseudotyped with BG505.W6M.C2 (A), ZM53M.PB12 (B), and JR-FL (C) Env clones. Infection was measured by flow cytometry as percentage of GFP-positive cells 48 h postinfection. Graphs show percentage of infected cells for wild-type (WT), L193A, and I423A Envs at a multiplicity of infection of 1. Bars represent the averages from three independent experiments. Error bars represent standard deviations.

Recent studies have suggested that Envs of most primary HIV-1 isolates exist in a closed conformation and only infrequently transit to downstream, more open conformations (11, 13, 18, 19). The ability of unliganded or CD4-bound Envs to transit to downstream states can affect viral tropism by allowing adaptability to cells with different CD4 levels (15, 16). Thus, we sought to determine how the conformational states of HIV-1 Envs affect the ability to utilize rhCD4 for viral entry. We took advantage of the fact that single amino acid substitutions in the gp120 domain of HIV-1 Env trimer can alter its conformational state (15, 16). For example, L193A substitution in the V1/V2 loop of gp120 allows Envs to populate the state 2 conformation. I423A substitution in the β20-β21 element of gp120 allows Envs to populate the state 3 conformation. We engineered L193A and I423A substitutions in three different Envs representing a T/F (BG505.W6M.C2), an acute-stage clone (ZM53M.PB12), and a chronic-stage isolate (JR-FL). For the two early-stage Envs, I423A substitution mediated entry in rhCD4_LOW_, rhCD4_HIGH_, and huCD4 cells with similar efficiencies, whereas the L193A substitution displayed lower efficiency of entry in rhCD4_LOW_ cells than in huCD4 and rhCD4_HIGH_ cells (Fig. 3A and B). As controls, we used the related wild-type (WT) Envs that utilized huCD4 efficiently but did not gain efficient entry even in cells that express high levels of rhCD4. The chronic-stage JR-FL Env was able to gain entry using both huCD4 and rhCD4—independent of Env conformational state or rhCD4 expression levels (Fig. 3C). Similar patterns of rhCD4 usage were observed at different multiplicities of infection (MOIs), indicating that the ability of open Env conformations to use physiological levels of rhCD4 is independent of virus input (see Fig. S1 in the supplemental material). Thus, our results suggest that single amino acid substitutions in Env that promote transitions to intermediate and open conformations allow more efficient usage of physiological levels of rhCD4 for viral entry.

10.1128/mBio.02752-21.1 FIG S1 Effect of virus input on rhCD4 usage by HIV-1 Envs in different conformations. Cf2Th/syn CCR5 cells expressing human or rhesus CD4 (indicated along the *x* axis) were infected with HIV-1_Q23ΔEnvGFP_ pseudotyped with BG505.W6M.C2 (A), ZM53M.PB12 (B), or JR-FL (C) Env clone. Infection was measured by flow cytometry as percentage of GFP-positive cells 48 h postinfection. Graphs show percentage of infected cells for wild-type (WT), L193A, and I423A Env variants at different multiplicities of infection (MOI; top panel, MOI = 0.1; bottom panel, MOI = 0.25). Bars represent the averages from three independent experiments. Error bars represent standard deviations. Download FIG S1, TIF file, 0.5 MB. Copyright © 2022 Vilmen et al. 2022 Vilmen et al. https://creativecommons.org/licenses/by/4.0/ This content is distributed under the terms of the Creative Commons Attribution 4.0 International license.

### Open Env conformations promote replication of SHIV-BG505 in rhesus lymphocytes.

**FIG 4:**
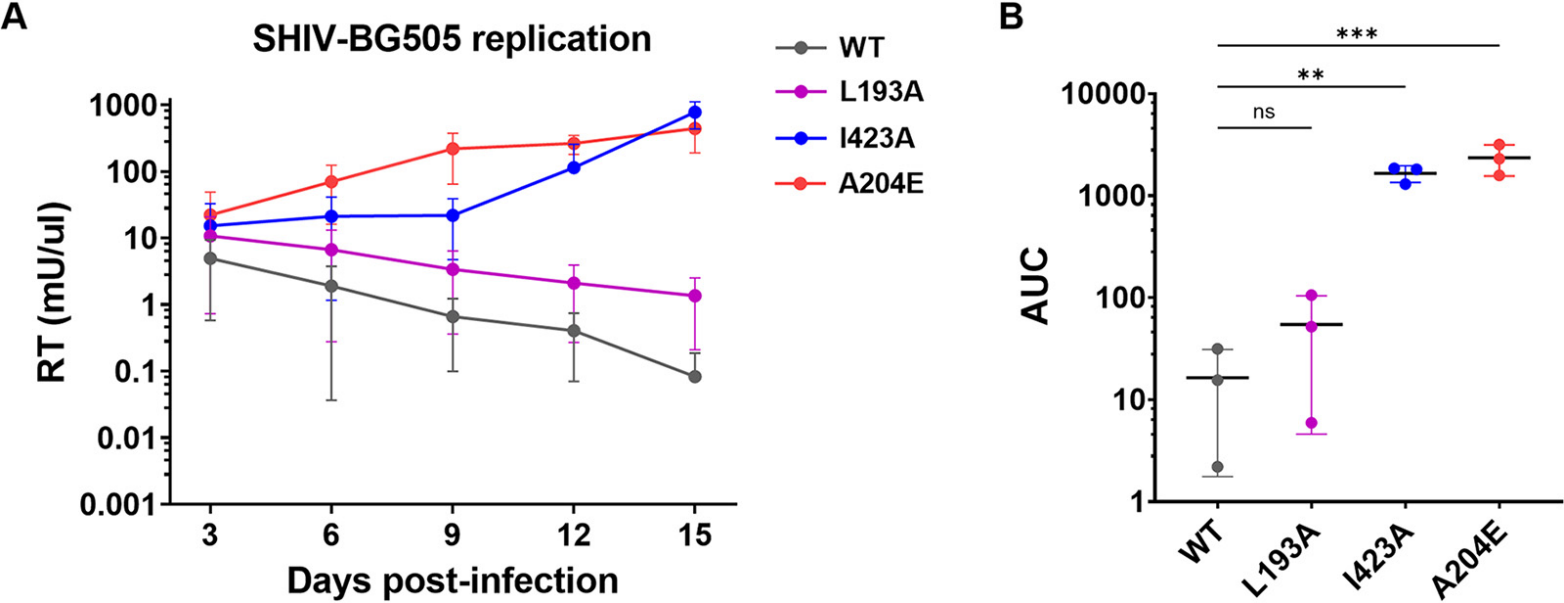
Effect of Env conformation on SHIV replication. (A) Replication kinetics of SHIV-BG505 variants in rhesus macaque 221 T lymphocytes over a 15-day time course. Reverse transcriptase (RT) activity in viral supernatants is plotted versus days postinfection. The key at the right of the graph indicates the identity of the Env variant (WT or indicated amino acid mutation) encoded by each SHIV. Each data point represents the average from three independent experiments, performed in duplicate. Error bars represent standard deviations. (B) Area under the curve (AUC) for indicated SHIV variants (*x* axis) determined from the replication curves shown in panel A. AUC values were compared to those for the SHIV-BG505 WT using one-way analysis of variance (ANOVA) followed by Dunnett’s multiple-comparison test. ***, *P = *0.0004; **, *P = *0.004. ns, not significant.

Next, we sought to determine whether improved rhCD4 usage by Envs that are in intermediate and open conformational states translate into enhanced replication of T/F SHIV in rhesus lymphocytes. For this purpose, we introduced the L193A or I423A substitution in the HIV-1_BG505_ T/F Envs encoded by SHIV-BG505. Importantly, we also included SHIV-BG505 with A204E substitution in the C1 region of gp120 as a benchmark control for replication. The A204E adaptive mutation, previously identified through serial passage of primary HIV-1 Envs in immortalized macaque CD4^+^ T lymphocytes (20), was sufficient to mediate viral entry in rhCD4_LOW_ cells at levels comparable to those in huCD4 cells (Fig. S2). More importantly, the A204E substitution induces conformational changes in the Envs that open up the Env trimers on the surface of virions (10), which likely explains its ability to utilize physiological levels of rhCD4. We measured the ability of these SHIVs to replicate in the immortalized rhesus macaque CD4^+^ T lymphocytes (21). As expected, the replication of SHIV encoding wild-type Envs (WT SHIV), which do not utilize rhCD4 efficiently, declined over the 15-day time course, indicating that it does not replicate in rhesus cells (Fig. 4). SHIVs encoding I423A or A204E substitutions replicated to significantly higher levels over the 15-day time course than WT SHIV. Interestingly, SHIV L193A, which encodes Env with an intermediate open conformation that allows it to use rhCD4 better than the WT Envs (Fig. 3A), did not display significantly improved growth kinetics compared to WT SHIV. Based on these findings, we conclude that open Env conformations, such as those conferred by I423A and A204E mutations, promote replication of SHIV-BG505 in rhesus lymphocytes.

10.1128/mBio.02752-21.2 FIG S2 Ability of HIV-1 Env with A204E substitution to use physiological levels of rhCD4 for viral entry. Cf2Th/syn CCR5 cells expressing human or rhesus CD4 (indicated along the *x* axis) were infected with HIV-1_Q23ΔEnvGFP_ pseudotyped with BG505 Env with the A204E mutation. Infection was measured by flow cytometry as percentage of GFP-positive cells 48 h postinfection. Graphs show percentage of infected cells at indicated MOI. Bars represent the averages from three independent experiments. Error bars represent standard deviations. Download FIG S2, TIF file, 0.2 MB. Copyright © 2022 Vilmen et al. 2022 Vilmen et al. https://creativecommons.org/licenses/by/4.0/ This content is distributed under the terms of the Creative Commons Attribution 4.0 International license.

### Open Env conformations promote CD4 binding and viral fusion.

**FIG 5:**
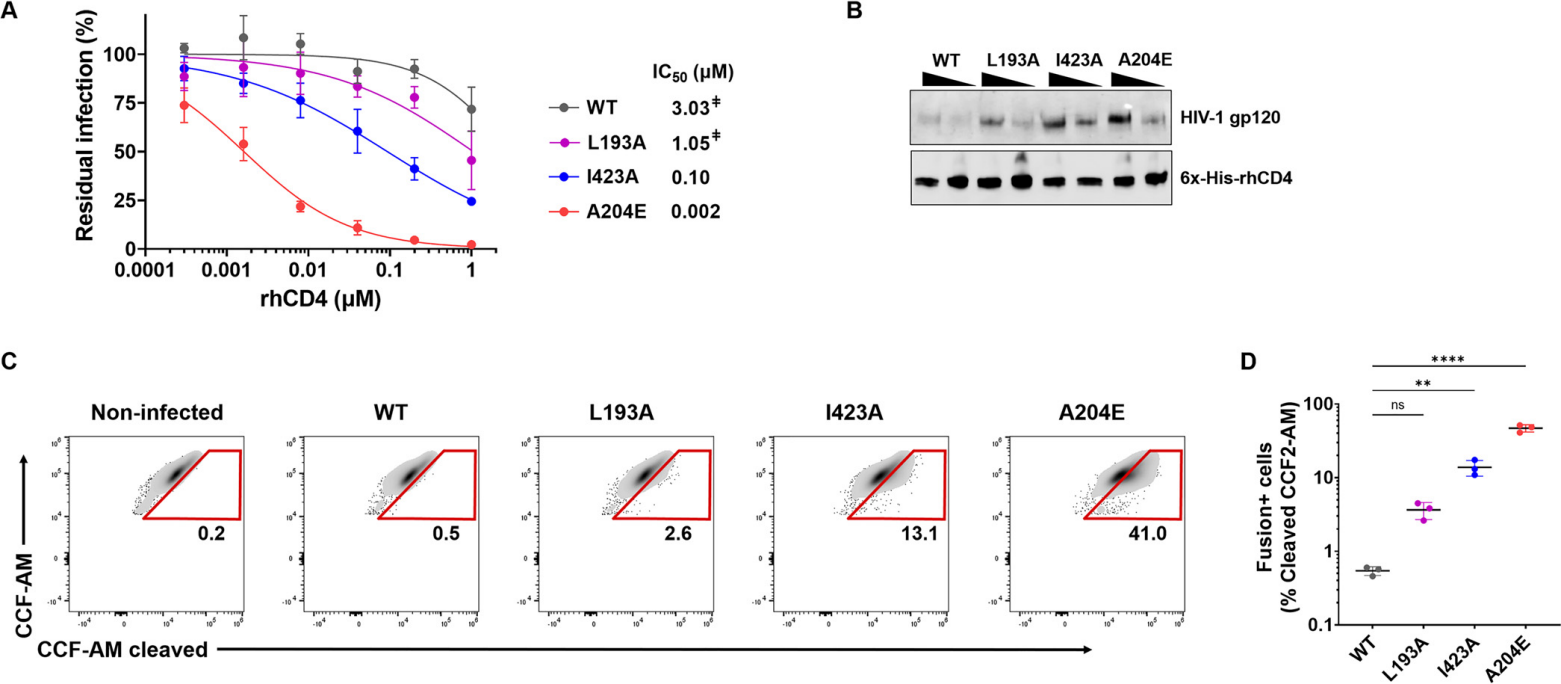
Effect of Env conformation on rhCD4 binding and viral fusion. (A) Sensitivity of SHIV-BG505 to neutralization by soluble rhCD4. Neutralization curves of the indicated SHIV variants were generated by plotting percent residual infection (*y* axis) against rhCD4 concentration (*x* axis). Each data point represents the average from two independent experiments, performed in duplicate. Error bars represent standard deviations. The calculated IC_50_s are shown. Where IC_50_ values were above the highest tested concentration, the extrapolated concentration is indicated by a double dagger. (B) Binding of SHIV-BG505 to rhCD4. Western blot analysis for affinity pulldown of His-tagged rhCD4 was performed with increasing amounts (400 and 800 mU of RT) of indicated SHIV virions. Immunoblotting was performed using anti-HIV-1 gp120 and anti-6×His antibodies. (C) Representative flow cytometry plots indicating percent viral fusion. Fusion of indicated SHIV-BG505 variants with rhesus macaque 221 T lymphocytes was measured as percentage of cells with cleaved CCF2-AM substrate. The identity of the Env variant (WT or indicated amino acid mutation) encoded by each SHIV is indicated above the plots. (D) Graph of percent viral fusion for indicated SHIV variants (*x* axis). Data represent the averages from three independent experiments, with individual data points shown as circles. Error bars represent standard deviations. Percent viral fusion was compared to that for the SHIV-BG505 WT using one-way ANOVA followed by Dunnett’s multiple-comparison test. ****, *P < *0.0001; **, *P = *0.0024.

Finally, we examined whether open Env conformations promote SHIV replication by enhancing rhCD4 binding and viral fusion. We evaluated the effects of Env conformation on rhCD4 binding by two independent approaches. First, we performed neutralization assays to measure the ability of soluble rhCD4 to bind functional Env trimers on SHIVs and compete with receptor binding to inhibit viral entry. WT SHIV was least sensitive to rhCD4 inhibition, indicating that it does not efficiently bind rhCD4 and interfere with receptor binding (Fig. 5A). SHIV L193A and SHIV I423A, which are stabilized in state 2 and state 3 Env conformations, respectively, were ∼3-fold and ∼30-fold more sensitive to rhCD4 inhibition than WT SHIV, suggesting that progressive opening of the Envs increases rhCD4 binding. SHIV A204E was most sensitive (∼1,500-fold) to rhCD4 inhibition, suggesting that it has the highest binding for rhCD4, likely attributable to “more open” conformation of its Env trimers. Similar neutralization trends, but with ∼2- to 10-fold more potent inhibition, were observed with soluble huCD4, indicating that HIV-1 Envs encoded by these SHIVs bind huCD4 with higher affinity than rhCD4 (Fig. S3A). Second, we employed a qualitative affinity pulldown assay to determine the binding of rhCD4 to Envs expressed on infectious SHIVs. Virions bound to His-tagged rhCD4 were affinity precipitated and their Env levels were measured by immunoblotting. Consistent with the results of the neutralization assay, progressive opening of the Env resulted in more virions binding to rhCD4, with highest binding observed in the case of SHIV A204E (Fig. 5B). Similar binding patterns were also observed when pulldowns were performed with huCD4 (Fig. S3B). Lastly, we investigated how Env conformation affects fusion of SHIVs with rhesus lymphocytes using a SHIV fusion assay. WT SHIVs, which did not efficiently bind rhCD4, were not fusogenic with the target cells (Fig. 5C and D). SHIV L193A and SHIV I423A, which are stabilized in state 2 and state 3 Env conformations, respectively, were ∼7-fold and ∼25-fold more fusogenic than WT SHIV, suggesting that fusogenicity increases with progressive opening of the Envs. SHIV A204E was the most fusogenic, with ∼87-fold-higher fusion than WT SHIV. Taken together, our results demonstrate that inducing open Env conformations, by introducing the I423A and A204E mutations, increases binding of Env to rhCD4 and subsequent SHIV-host membrane fusion.

10.1128/mBio.02752-21.3 FIG S3 Effect of Env conformation on huCD4 binding. (A) Sensitivity of SHIV-BG505 to neutralization by soluble huCD4. Neutralization curves of the indicated SHIV variants were generated by plotting percent residual infection (*y* axis) against huCD4 concentration (*x* axis). The key at the right of the graph indicates the identity of Env variant (wild-type [WT] or indicated amino acid mutation) encoded by each SHIV. Each data point represents the average from two independent experiments, performed in duplicate. Error bars represent standard deviations. The calculated IC_50_s are shown. Where IC_50_ values were above the highest tested concentration, the extrapolated concentration is indicated by a double dagger. (B) Binding of SHIV-BG505 to huCD4. Western blot analysis for affinity pulldown of His-tagged huCD4 was performed with increasing amounts (400 and 800 mU of RT) of indicated SHIV virions. Immunoblotting was performed using anti-HIV-1 gp120 and anti-6×His antibodies. Download FIG S3, TIF file, 0.4 MB. Copyright © 2022 Vilmen et al. 2022 Vilmen et al. https://creativecommons.org/licenses/by/4.0/ This content is distributed under the terms of the Creative Commons Attribution 4.0 International license.

## DISCUSSION

Here, we provide first genetic evidence that conformational state of HIV-1 Envs governs rhCD4 usage. Using HIV-1 Envs with single amino acid substitutions that altered their conformational states, we demonstrated that transitions to intermediate and open Env conformations allow usage of physiological levels of rhCD4 for viral entry, which is otherwise a suboptimal receptor for entry. Moreover, by introducing these changes into the isogenic SHIV backbone, we found that open Env conformations promote rhCD4 binding, fusion-mediated cell entry, and replication in rhesus lymphocytes.

Thermodynamically, most primary HIV-1 Envs are in a high-free-energy, closed conformation. Single-molecule fluorescence resonance energy transfer studies have demonstrated that either spontaneously or upon CD4 binding, Env transitions from closed (state 1) to open (state 3) conformation through a functional intermediate (state 2) (11). Multiple amino acid residues restrain Env in state 1 and CD4 binding triggers Env transitions from state 1 to downstream, lower-energy states (15, 16, 22–25). For example, L193A and I423A substitutions release the state 1 restraints and stabilize Env in state 2 and state 3 conformations, respectively. Our findings of improved rhCD4 usage by Envs bearing L193A and I423A substitutions suggest that transitions to intermediate and open conformations advance HIV-1 Env on the entry pathway and likely facilitate Env engagement with a suboptimal rhCD4 receptor. Although Envs with L193A substitutions, which are in intermediate open conformations, utilized rhCD4 better than WT Env, SHIV encoding the L193A substitution did not display significantly improved viral fusion or replication. In contrast, SHIVs encoding Envs with I423A and A204E substitutions, which are in more open conformations, displayed significantly increased rhCD4 engagement, viral fusion, and replication. One possible explanation for the differences in growth kinetics of SHIVs encoding L193A and I423A substitutions could be the ∼2- to 3-fold-lower infectivity of L193A Envs than I423A Envs. During spreading infection in rhesus lymphocytes, which involves multiple rounds of infection over a 15-day time course, the differences in absolute infectivity are likely amplified over time and are reflected in robust differences in viral growth kinetics. Overall, our results indicate that open Env conformations conferred by I423A and A204E mutations promote rhCD4 binding, viral fusion, and replication of SHIV in rhesus lymphocytes. Collectively, these findings suggest that modulating the conformational dynamics of viral Env by altering selected amino acid residues can help overcome the cross-species entry barrier, which, in turn, facilitates replication and adaptation of the virus in a new host species.

Our findings suggest that downstream, open Env conformations facilitate usage of rhCD4 and highlight that most T/F and early-stage HIV-1 Envs have an inherently low propensity to use rhCD4. For instance, only one (subtype D Env 191859) of the eight early-stage Envs tested was able to use physiological levels of rhCD4 for viral entry. Testing engineered I423A substitution in 191859 Env resulted in ∼2- to 3-fold improvement in usage of physiological levels of rhCD4 for viral entry (Fig. S4), suggesting that opening of the Env improves the efficiency of rhCD4 usage. Taken together, our findings explain why development of SHIVs encoding T/F and early-stage Envs has been challenging and often requires extensive adaptation in human and/or rhesus lymphocytes—a process that typically selects for open Env conformation (2, 10, 20). Consistent with this notion, SHIV-BG505 encoding A204E adaptive mutation, previously identified through *in vitro* evolution experiments (20), displayed the highest replication in rhesus lymphocytes, viral-host membrane fusion, and sensitivity to soluble CD4. These findings also offer an explanation as to why some existing pathogenic SHIVs derived using primary HIV-1 Envs display high sensitivity to soluble CD4 (26–28), which correlates with open Env conformation in which the CD4 binding site is exposed.

10.1128/mBio.02752-21.4 FIG S4 Ability of subtype D Env 191859 with the I423A substitution to use physiological levels of rhCD4 for viral entry. Cf2Th/syn CCR5 cells expressing human or rhesus CD4 (indicated along the *x* axis) were infected with HIV-1_Q23ΔEnvGFP_ pseudotyped with 191589 Env with the I423A mutation. Infection was measured by flow cytometry as percentage of GFP-positive cells 48 h postinfection. Graphs show percentage of infected cells at indicated MOI. Bars represent the averages from three independent experiments. Error bars represent standard deviations. Download FIG S4, TIF file, 0.2 MB. Copyright © 2022 Vilmen et al. 2022 Vilmen et al. https://creativecommons.org/licenses/by/4.0/ This content is distributed under the terms of the Creative Commons Attribution 4.0 International license.

In support of our findings that open Env conformations facilitates rhCD4 usage, prior studies have determined the effects of single amino acid substitutions on Env conformational state by measuring sensitivity to broadly neutralizing antibodies (bNAbs) and sCD4. L193A and I423A substitutions in three different Envs representing a T/F (BG505.W6M.C2), an acute-stage clone (ZM53M.PB12), and a chronic-stage isolate (JR-FL) displayed neutralization profiles that are expected for open Env conformations (15, 16). For example, all three Envs with L193A or I423A substitution were more sensitive to state 2/3-preferring sCD4 and 4E10 bNAb and more resistant to state 1-preferring PG9 and VRC03 bNAbs than the related wild-type Envs. In a separate study, A204E substitution in Env conferred resistance to bNAbs directed to quaternary epitopes and sensitivity to antibodies directed to internal epitopes in the V2/V3 Env domains, suggesting that the A204E Env trimer adopts a more open conformation (10). Consistent with this notion, the T/F BG505 Env is ∼40-fold more resistant to sCD4 inhibition than chronic-stage JR-FL Env (15, 16). Moreover, BaL Envs are consider tier 1B Envs, and they are sensitive to several antibodies that recognize internal epitopes, such as 830A and 447-52D (CATNAP [https://www.hiv.lanl.gov/components/sequence/HIV/neutralization/index.html]). Thus, the conformation of BaL Envs is probably more open than the Env conformation of BG505 and JR-FL.

Our study also suggests that additional parameters may contribute to efficient infection of rhCD4-expressing target cells. Notably, both JR-FL and BaL Envs efficiently use rhCD4 for entry but may represent different Env conformational states. Thus, additional factors, such as affinity of binding to the rhCD4 and hCD4 receptors, can play a role in the entry process, as has been previously reported (29). Overall, it is likely that the intrinsic conformational state of each Env trimer defines the ability to infect cells expressing rhCD4 and this ability can be further modified by opening or closing the specific HIV-1 Envs. From the perspective of transmission, it might be favorable for the virus to adopt a more closed Env conformation. This is because most neutralizing and antibody-dependent cellular cytotoxicity antibodies in the patients’ sera are directed against open Env conformation, and therefore, T/F viruses are likely under selective pressure to maintain Env in a closed conformation to evade host humoral responses (18, 19, 30, 31).

In summary, our findings have helped identify a key parameter for future design of SHIVs: the conformational state of Env. Considerable efforts are being made to engineer SHIVs that encode circulating HIV-1 Env, utilize rhCD4 for entry, replicate in rhesus macaques without extensive adaptation, and retain as much of the biological characteristics of HIV-1 Envs as possible (32–34). Thus, it will be of interest to identify and define the minimal open conformational state of HIV-1 Env that affords usage of rhCD4 and replication of SHIVs in rhesus macaques with minimal impact on Env trimer structure and antigenic profile. Based on the findings of this study, the following considerations could be useful for SHIV design: (i) selection of circulating HIV-1 Env with high inherent propensity to sample downstream, open conformations and (ii) engineering changes in Env that lower the free energy needed for transition to open conformations without significantly altering its antigenicity.

## MATERIALS AND METHODS

### Cells, envelope clones, plasmids, and proteins.

HEK293T (ATCC CRL-3216), HeLa TZM-bl (35) (NIH AIDS Reagent program catalog no. 8129), and Cf2Th/syn CCR5 (36) (NIH AIDS Reagent Program; catalog no. 4662) cells were cultured in Dulbecco’s modified Eagle medium (DMEM; Gibco) supplemented with 10% fetal bovine serum (FBS; Sigma), 2 mM l-glutamine (Gibco), and 1× penicillin-streptomycin (Gibco) (complete DMEM). Cf2Th/syn CCR5 cells, which are engineered to express human CCR5, were further supplemented with 400 μg/mL of Geneticin (Gibco) to maintain CCR5 expression. Immortalized rhesus macaque 221 T lymphocytes (21) were cultured in Iscove’s modified Dulbecco’s medium (IMDM) supplemented with 10% FBS, 2 mM l-glutamine, 1× penicillin-streptomycin, and 100 U/mL of interleukin-2 (Roche) (complete IMDM). Rhesus macaque peripheral blood mononuclear cells (PBMCs) were isolated from whole blood (Washington National Primate Research Center) from two independent donors using human erythrocyte lysing kit (R&D Systems) and 95% Lymphoprep (Stemcell Technologies) following the manufacturer’s protocols.

The following Env clones from early HIV-1 infections were used: BG505.W6M.C2, Q23.17, TRO.11, CAAN5342.A2, Du422.1, ZM53M.PB12, QB857.110I.B3, and 191859. As a control, two Env clones (JR-FL and BaL.01) of chronic HIV-1 strains were used. The following Env clones encoding the L193A, I423A, and A204E mutations were used: BG505.W6M.C2, ZM53M.PB12, and JR-FL.

A β-lactamase (BlaM) gene fused to the N terminus of SIVmac239 Vpr (GenBank accession no. M33262) separated by a six-glycine–one-lysine linker was synthesized as a DNA fragment (Integrated DNA Technologies), digested, and ligated into pcDNA3.1 (Invitrogen) using KpnI and NotI restriction sites to generate the pcDNA3.1-BlaM-SIVmac239 Vpr plasmid. All plasmids generated in this study were verified by Sanger DNA sequencing.

Soluble rhCD4 (Met1-Trp390; catalog no. 90274-C08H) and huCD4 (Met 1-Trp 390; catalog no. 10400-H08H) proteins with C-terminal 6×His tags were purchased from Sino Biological.

### Pseudovirus and SHIV production.

Green fluorescent protein (GFP) reporter pseudoviruses were generated as described previously (37). Briefly, HEK293T cells were cotransfected with 4 μg of Env-deficient HIV-1 proviral plasmid (Q23ΔEnvGFP) and 2 μg of the HIV-1 Env clone of interest using Fugene 6 transfection reagent (Roche) following the manufacturer’s protocol. Replication-competent SHIV stocks were generated as described previously (38). The viral titer of each pseudovirus and SHIV stock was determined by infecting TZM-bl cells and staining for β-galactosidase activity 48 h postinfection (35).

### Generation of stable cell lines.

Cf2Th/syn CCR5 cells stably expressing rhCD4 were generated using methods described previously (39). Briefly, retroviral pseudoviruses were generated in HEK293T cells by cotransfecting pLPCX-rhCD4 (retroviral vector encoding rhCD4), pJK3 (murine leukemia virus [MLV]-based packaging plasmid), and pMD.G (vesicular stomatitis virus glycoprotein [VSV-G] plasmid) at a ratio of 1:1:0.5 using Fugene 6 transfection reagent following the manufacturer’s protocol. Forty-eight hours posttransfection, the pseudoviruses were concentrated and used to transduce 10^5^ Cf2Th/syn CCR5 cells. Transduced cells were cultured in complete DMEM supplemented with 400 μg/mL of Geneticin (to maintain CCR5 expression) and 2 μg/mL of puromycin (to select for CD4 expression; Sigma). The drug-selected cells with low (rhCD4_LOW_) or high (rhCD4_HIGH_) levels of rhCD4 expression were obtained by sorting the cells on a FACSAria II cell sorter (BD Biosciences) using an allophycocyanin (APC)-conjugated CD4 monoclonal antibody (BD Biosciences; catalog no. 551980) using a previously described CD4 staining method (17). The rhCD4_LOW_ cells were sorted by gating on the CD4-expressing cell population that overlapped with the CD4-stained rhesus macaque PBMCs. The rhCD4_HIGH_ cells were sorted by gating on the top 20% of the CD4-expressing cell population. Cf2Th/syn CCR5 cells stably expressing huCD4 have been described previously (39).

### CD4 infectivity assay.

Infection of Cf2Th/syn CCR5 cells expressing huCD4, rhCD4_LOW_, and rhCD4_HIGH_ with GFP reporter pseudoviruses was performed as described previously (39). Briefly, cells were infected at multiplicities of infection (MOIs) of 0.1, 0.25, and 1 in the presence of 10 μg/mL of DEAE-dextran by spinoculation at 1,200 × *g* for 90 min. After 48 h, cells were harvested, fixed in 2% paraformaldehyde, washed twice, and resuspended in fluorescence-activated cell sorter (FACS) buffer (1× phosphate-buffered saline [PBS], 1% FBS, 1 mM EDTA). Cells were analyzed for GFP expression on an Attune NxT flow cytometer (Life Technologies). The data from ∼10^4^ cells were analyzed using FlowJo version 10.7.1.

### Construction of SHIV proviral clones.

Full-length proviral SHIV plasmid encoding BG505.W6M.B1 Env (40) with the A204E mutation (SHIV-BG505 A204E) has been described previously (41). The EcoRV-MfeI region of BG505.W6M.B1 Env (wild type [WT]) and its L193A and I423A variants were synthesized as DNA fragments (Twist Biosciences). SHIV-BG505 WT, SHIV-BG505 L193A, and SHIV-BGB505 I423A proviral clones were generated by digesting and ligating the DNA fragments into the SHIV-BG505 A204E proviral plasmid using EcoRV and MfeI restriction sites. The generated SHIV proviral plasmids were verified by DNA sequencing.

### SHIV replication time course.

Replication of SHIVs was assessed as described previously (38). Briefly, 4 × 10^6^ 221 T lymphocytes were infected at an MOI of 0.02 by spinoculation at 1,200 × *g* for 90 min at room temperature. After spinoculation, cells were washed four times with 1 mL of complete IMDM, resuspended in 5 mL of complete IMDM, and plated in one well of a six-well plate. Every 3 days, two-thirds of the cultures was harvested and replenished with fresh, complete IMDM. Viral supernatants were collected from the harvested cultures by pelleting at 650 × *g* for 5 min at room temperature. Reverse transcriptase (RT) activity in viral supernatants was measured using the RT activity assay.

### SHIV neutralization assay.

Neutralization of SHIVs with rhCD4 and huCD4 were performed as described previously (16) but using TZM-bl target cells. The calculated half-maximal inhibitory concentration (IC_50_) values represent the soluble CD4 micromolar concentration at which 50% of the virus was neutralized.

### CD4-SHIV binding assay.

Affinity pulldown assays were performed using Ni Sepharose 6 Fast Flow resin (Cytiva Lifesciences), His-tagged rhCD4 or huCD4, and SHIV stocks to assess binding of CD4 to SHIVs. Twenty microliters of Ni resin was equilibrated in binding buffer (50 mM HEPES [pH 7.5], 250 mM NaCl, 50 mM imidazole, and 2 mM β-mercaptoethanol) by washing three times (10,000 × *g* for 1 min) with 100 μL of binding buffer. Equilibrated Ni resin was incubated for 10 min with 1 μg of His-CD4 in 20 μL of binding buffer. Binding reactions were set up by adding equal amounts of indicated SHIV virions (equivalent to 800 and 400 mU of RT) in 160 μL of binding buffer and incubated for 1 h at room temperature, under rotation. Reaction mixtures were then washed three times (10,000 × *g* for 1 min) with 100 μL of binding buffer to remove unbound CD4/virions. The resulting CD4-virion complexes bound to the Ni resin were extracted using 4× Laemmli sample buffer (Bio-Rad), heated at 95°C for 5 min, and subjected to SDS-PAGE/Western blotting. Standard Western blotting procedures were used with the following antibodies: HIV-1 gp120 (NIH AIDS Reagent Program; catalog no. 288) and 6×His tag (Thermo Fisher; catalog no. MA1-21315-HRP).

### SHIV fusion assay.

The HIV-1 BlaM-Vpr virus fusion assay described previously (42, 43) was modified to evaluate SHIV fusion. Briefly, BlaM-Vpr was modified by swapping the HIV-1 Vpr with the SIVmac239 Vpr to retain the cognate Gag p6-SIVmac Vpr interaction necessary for efficient virion incorporation of Vpr (44). SHIVs containing BlaM-SIVmac239 Vpr fusion protein were generated by cotransfecting HEK293T cells with 4.5 μg of proviral SHIV plasmid and 1.5 μg of pcDNA3.1-BlaM-SIVmac239 Vpr plasmid using Fugene 6 transfection reagent following the manufacturer’s protocol. Forty-eight hours posttransfection, virus-containing supernatant was harvested, passed through a 0.2-μm sterile filter, concentrated ∼10-fold using Amicon Ultracel 100-kDa filters (Millipore), aliquoted, and stored at −80°C. RT activity of viral stocks was measured using the RT activity assay. A total of 10^5^ 221 T lymphocytes in 100 μL of complete IMDM were infected with BlaM-SIVmac239 Vpr-containing SHIVs equivalent to 720 mU of RT by spinoculation at 1,200 × *g* for 90 min, followed by incubation at 37°C and 5% CO_2_ for 1 h. Fusion-mediated SHIV entry was quantified by monitoring the conversion of fluorescent BlaM CCF2-AM substrate dye. After infection, cells were washed once with 300 μL of cold CO_2_-independent medium (Gibco) without FBS and resuspended in 100 μL of CO_2_-independent medium supplemented with 10% FBS. Cells were incubated with CCF2-AM substrate (LiveBLAzer CCF2-AM kit; Invitrogen) following the manufacturer’s protocol in the presence of 1.8 mM probenecid (Sigma) for 2 h. Cells were washed three times with 300 μL of cold CO_2_-independent medium without FBS and once with 1× PBS, fixed with 200 μL of 2% paraformaldehyde, washed once with 1× PBS, and resuspended in 400 μL of FACS buffer. The fluorescence of cleaved CCF2-AM substrate was measured on an Attune NxT flow cytometer and data were analyzed using FlowJo version 10.7.1.

### Reverse transcriptase activity assay.

RT activity assay was performed as described previously (43, 45), with minor modifications. Briefly, 5 μL of viral supernatant, viral stock, or RT standard was lysed in 5 μL of 2× lysis buffer (100 mM Tris HCl [pH 7.4], 50 mM KCl, 0.25% Triton X-100, 40% glycerol) in the presence of 4 U of RNaseOUT (Invitrogen) for 10 min at room temperature. Viral lysate was diluted 1:10 by adding 90 μL of nuclease-free water (Life Technologies). Reverse transcriptase quantitative PCRs (qRT-PCRs) were prepared by mixing 9.6 μL of diluted viral lysate with 10.4 μL of reaction mixture containing 10 μL of 2× Maxima SYBR green/ROX qPCR master mix (Thermo Fisher), 0.1 μL of 4-U/μL RNaseOUT, 0.1 μL of 0.8-μg/μL MS2 RNA template (Roche), and 0.1 μL each of 100 μM forward (5′-TCCTGCTCAACTTCCTGTCGAG-3′) and reverse (5′-CACAGGTCAAACCTCCTAGGAATG-3′) primers. qRT-PCR was performed using a QuantStudio 3 real-time PCR machine (Applied Biosystems). Viral titers were calculated from a standard curve generated using recombinant reverse transcriptase (Millipore; catalog no. 382129).
